# A two-center retrospective study: association of early caffeine administration and oxygen radical diseases in neonatology in Chinese preterm neonates

**DOI:** 10.3389/fped.2023.1158286

**Published:** 2023-06-14

**Authors:** Huiqing Ye, Liyang Bai, Manting Yang, Xiaoyuan Yang, Maofei Zheng, Xiaobing Zhong, Lifen Yang, Zhuanggui Chen, Xinqi Zhong

**Affiliations:** ^1^ Department of Neonatology, The Third Affiliated Hospital of Guangzhou Medical University, Guangzhou, China; ^2^ Department of Pediatrics, The Third Affiliated Hospital of SunYat-sen University, Guangzhou, China; ^3^ Guangdong Provincial Key Laboratory of Major Obstetric Diseases, Guangzho, China

**Keywords:** caffeine treatment, oxygen radical diseases in neonatology, preterm, retrospective study, receiver operating characteristic curves

## Abstract

**Introduction:**

Since December 2012, the prophylactic use of caffeine to treat AOP in preterm infants has been approved in China. This study aimed to investigate the relationship between early caffeine treatment initiation and the incidence of oxygen radical diseases in neonatology (ORDIN) in Chinese preterm infants.

**Methods:**

A retrospective study was conducted at two hospitals in South China, involving 452 preterm infants with gestational ages less than 37 weeks. The infants were divided into early (227 cases, initiating within 48 h after birth) and late (225 cases, initiating over 48 h after birth) caffeine treatment group. Logistic regression analysis and Receiver Operating Characteristic (ROC) curves were used to evaluate the association between early caffeine treatment and the incidence of ORDIN.

**Results:**

The results showed that extremely preterm infants in early treatment group had a lower incidence of PIVH and ROP compared to those in the late treatment group (PIVH, 20.1% versus 47.8%, *P* = 0.02; ROP, 70.8% versus 89.9%, *P* = 0.025). Very preterm infants in the early treatment group had a lower incidence of BPD and PIVH compared to those in the late treatment group (BPD, 43.8% versus 63.1%, *P* = 0.002; PIVH, 9.0% versus 22.3%, *P* = 0.001). Moreover, VLBW infants who received early caffeine treatment exhibited a decreased incidence of BPD (55.9% versus 80.9%, *P* = 0.000), PIVH (11.8% versus 33.1%, *P* = 0.000), and ROP (69.9% versus 79.8%, *P* = 0.043) compared to those in the late treatment group. Infants in the early caffeine treatment showed a reduced likelihood of PIVH (adjusted odds ratio, 0.407; 95%CI, 0.188–0.846) but did not exhibit a significant association with other terms of ORDIN. ROC analysis revealed that early initiation of caffeine treatment was associated with lower risk of BPD, PIVH, and ROP in preterm infants.

**Discussion:**

In conclusion, this study demonstrates that early initiation of caffeine treatment is associated with a decreased incidence of PIVH in Chinese preterm infants. Further prospective investigations are necessary to verify and elucidate the precise effects of early caffeine treatment on complications in preterm Chinese infants.

## 1. Introduction

In recent years, the progress of perinatal medicine, including the availability of neonatal intensive care units and mechanical ventilators and the administration of antenatal steroids and surfactants, has led to increased survival rates in preterm infants, particularly extremely preterm infants. However, due to their immature organ development, extremely or very preterm infants are at high risk for preterm-related diseases in various organs or systems, including bronchopulmonary dysplasia (BPD), periventricular–intraventricular hemorrhage (PIVH) (1), periventricular leukomalacia (PVL), retinopathy of prematurity (ROP) (2), and necrotizing enterocolitis (NEC). These terms of diseases are defined as oxygen radical diseases in neonatology (ORDIN), the concept of which was raised by Saugstad in 1988 (3). ORDIN is a complex and multifactorial disease with a common pathological mechanism involving free radical damage. Currently, there are limited therapeutic strategies for preventing ORDIN, and caffeine and non-steroidal anti-inflammatory drugs are being considered as potential candidates for improving neonatal outcomes.

Methylxanthines have been used for several decades to prevent apnea of prematurity (AOP) in preterm infants. Caffeine is one of the most common methylxanthines widely used in neonatal intensive care units (NICU) due to its longer-lasting effects, greater safety, and lower cost compared to other methylxanthines. Studies have shown that caffeine significantly reduced the duration of mechanical ventilation and the incidence of BPD, intraventricular hemorrhage (IVH), and other terms of ORDIN (4–6). The protective effects of caffeine on ORDIN are attributed to its various biological activities, such as acting as an antagonist of adenosine receptors (AR) and exhibiting antioxidant effects (7).

The timing and dose of caffeine administration can have varying effects on preterm infants, and there is currently no consensus on standardized protocols for caffeine treatment till now in the NICU (6). Early initiation of caffeine therapy is generally recommended, but the definition of “early” can vary between studies. Previous studies showed that early use of caffeine within 48 h or 72 h after birth had a lower incidence of AOP, BPD, patent ductus arteriosus (PDA), rates of death, and IVH in preterm infants compared to late use (8–10). However, a study on preterm infants ≤32 weeks of gestation age found no significant difference in BPD incidence between early and late caffeine treatment, although early treatment with caffeine significantly reduced the length of duration of mechanical ventilation, invasion ventilation rate, and the risk of incidence of IVH (11). The variability in outcomes of caffeine treatment may be attributed to various factors such as ethnic background, gestation age, initial treatment timing, and dose of caffeine. The effects of caffeine treatment on different preterm infants (extremely preterm, very preterm, and moderate and late preterm) remain unclear.

Compared to the long history of caffeine application in preterm infants, the use of caffeine for the management of AOP has only been approved in China since December 2012. There is a shortage of research on the timing and outcomes of caffeine treatment for ORDIN. In this study, data from two hospitals in Guangzhou, China, were retrospectively analyzed to investigate the association between early treatment of caffeine and outcomes of ORDIN in different subgroups of preterm infants.

## 2. Method

### 2.1. Study design and subjects

We conducted a retrospective study of those preterm neonates who were admitted to the Neonatology Department at the Third Affiliated Hospital of Guangzhou Medical University and the Third Affiliated Hospital of Sun Yat-sen University between January 2017 and December 2019. The study subjects were enrolled based on the following inclusion criteria: (1) gestational age (GA) less than 37 weeks at birth; (2) birth weight (BW) less than 2,500 g; (3) admission to the hospital within 24 h after birth; and (4) receiving caffeine citrate during hospitalization. Neonates who met any of the following exclusion criteria were excluded from the study: (1) death within 24 h after admission; (2) severe congenital malformation; and (3) *t* inherited metabolic diseases.

The eligible preterm neonates were divided into two groups: the early caffeine treatment group (ECT), comprising those who received caffeine citrate within 48 h after birth (*n* = 227), and the late caffeine treatment group (LCT), comprising those who received caffeine citrate over 48 h after birth (*n* = 225). Caffeine treatment was administered in two scenarios: (1) Immediate use following an episode of apnea, which is defined as the cessation of airflow for more than 20 s accompanied by a heart rate of <100 beats per minute, cyanosis, or a decrease in oxygen saturation. The decision to initiate caffeine treatment was made by clinicians or nurses accordingly. (2) Prior to the removal of mechanical ventilation during extubation, all preterm infants received a loading dose of caffeine citrate (20 mg/kg) through intravenous drip for 30 min, followed by a maintenance dose of 5–10 mg/kg daily, which started 24 h after the loading dose. Caffeine administration continued until the apnea symptoms disappeared. Both groups received the same routine treatments, including electrocardiograph monitoring, being kept in a warm environment, and keeping the airway open. The duration of caffeine treatment in earliest completion time (ECT) and latest completion time (LCT) were 16.7 ± 12.5 days and 17.0 ± 13.8 days, respectively, with no significant difference (*P *> 0.05).

### 2.2. Definitions and diagnostic criteria

The diagnosis of birth asphyxia in this study was based on the criteria established by the experts' consensus on the diagnosis and grading of neonatal asphyxia in China (12): (1) the presence of high-risk factors that lead to asphyxia; (2) inability to establish effective spontaneous breathing after birth and an Apgar score ≤7 at 1 or 5 min after birth; (3) umbilical artery blood gas analysis with pH < 7.15; and (4) exclusion of other conditions that might cause low Apgar scores, such as respiratory, circulatory, and central nervous system congenital malformations, neuromuscular disorders, fetal hypovolemic shock, fetal edema, the use of high-dose anesthetic analgesics or magnesium sulfate during labor, and fetal passive drug poisoning. Items 2–4 are mandatory indicators, while item 1 is a reference indicator.

Preeclampsia was diagnosed when there is an elevation in blood pressure and the presence of proteinuria (excess protein in the urine) after 20 weeks of pregnancy, along with accompanying symptoms such as headaches, dizziness, nausea, vomiting, and upper abdominal discomfort. Eclampsia is diagnosed when individuals with preeclampsia develop more severe symptoms, including seizures or a state of coma. Fetal growth retardation, also referred to as intrauterine growth restriction (IUGR), was diagnosed through the use of fetal ultrasound. Fetal distress is a recognized term utilized to denote the occurrence of hypoxia in the uterine environment. It was determined through observed changes in fetal heart rate, anomalous fetal movements, and alterations in the composition of amniotic fluid. Premature rupture of membranes was diagnosed when there is a spontaneous rupture of the fetal membranes prior to the onset of labor or delivery. Gestational diabetes mellitus refers to the condition in which normal glucose metabolism or impaired glucose tolerance is present before pregnancy or develops and was diagnosed during pregnancy. Cholestasis is characterized by pruritus of the skin without apparent lesions during the later stages of pregnancy, occasionally accompanied by mild jaundice and limited additional symptoms. Serum biochemical markers reveal a mild to moderate elevation in transaminase and bilirubin levels, as well as an increase in glycocholic acid. The diagnosis of acute chorioamnionitis involves the identification of inflammation caused by the infiltration of pathogens in the chorionic and amniotic membranes of the placenta. Placenta previa is defined as the abnormal attachment of the placenta to the lower segment of the uterus after 28 weeks of pregnancy, with the possibility of the placenta's lower edge reaching or partially covering the cervical opening, which is positioned lower than the fetal presentation. Placental abruption was defined as the partial or complete separation of the normally located placenta from the uterine wall, which occurs after 20 weeks of pregnancy or during the delivery process, before the fetus is expelled. Antepartum hemorrhage is defined as the occurrence of vaginal bleeding after 28 weeks of pregnancy. Vaginitis is defined by the presence of external symptoms in the vaginal area, such as itching, burning pain, irritation, and abnormal vaginal discharge. Neonatal respiratory distress syndrome is characterized by acute respiratory failure resulting from extensive alveolar collapse, lung damage, and exudation in both lungs, which can be attributed to an insufficient production of pulmonary surfactant. Neonatal infection is defined as the presence of pathogenic microorganisms causing infection in newborns. Intrauterine infection refers to the transmission of pathogens from an infected pregnant woman to the fetus through the placenta during pregnancy. Neonatal infectious pneumonia encompasses pneumonia acquired prior to birth, during the birthing process, or after birth. Neonatal sepsis is defined as a systemic inflammatory response resulting from the invasion, growth, reproduction, and toxin production of pathogens within the bloodstream of newborn infants. Pulmonary arterial hypertension in neonates is characterized by the sustained elevation of pulmonary vascular resistance following birth. Fetal arterial catheterization enables blood circulation, but after birth, as the lungs expand and assume gas exchange function, the catheters naturally close. If the ductus arteriosus remains open, it is known as patent ductus arteriosus. Intracerebral hemorrhage refers to the rupture of cerebral arteries, veins, or capillaries resulting in bleeding within the brain tissue. Asphyxia refers to the condition in which a newborn is unable to initiate normal spontaneous breathing after birth, resulting in hypoxemia, hypercapnia, and potential damage to multiple organs. Small for gestational age (SGA) infants are defined as newborns whose birth weight falls below the 10th percentile of the average birth weight for infants of the same gestational age.

For the assessment of patent ductus arteriosus (PDA), a routine cardiac ultrasound examination was conducted within one week of birth and prior to discharge. However, if clinical manifestations such as apnea, increased oxygen or ventilator requirements, metabolic acidosis, tachycardia, heightened precordial pulsation, hypotension, an increased pulse pressure difference (>25 mmHg), or the presence of murmurs were observed, an immediate cardiac ultrasound examination was performed.

Neonatal infection encompasses various types of infections, such as sepsis, intrauterine infection, infectious pneumonia, and others (e.g., neonatal omphalitis). Diagnostic criteria for sepsis include the isolation of pathogenic bacteria from blood culture or sterile body cavities. Clinical manifestations, along with specific examination results or positive pathogen antigen/DNA detection in blood samples, are used for diagnosis.

The diagnosis of ORDIN was performed according to Practical Neonatology (fifth version). Preterm infants requiring oxygen therapy for 28 days or longer were diagnosed with BPD. ROP was diagnosed and classified by an ophthalmologist according to the International Classification of Retinopathy of Prematurity (ICROP). PIVH and PVL were diagnosed based on the results of craniocerebral ultrasound or MRI and were graded according to the Volpe method (2008) and De-Vries criteria, respectively. The diagnosis of NEC was based on symptoms such as abdominal distension, hematochezia, and absence of bowel sound, as well as x-ray images.

### 2.3. Data collection and statistical analysis

The study collected baseline demographics and clinical information of both mothers and infants, including the gender of preterm infants, gestational age, BW, maternal age, delivery mode, antenatal steroid treatments, and perinatal conditions (Supplementary Table S1). Additionally, the outcomes of caffeine treatment in both groups were collected.

The demographic and clinical characteristics of both the maternal and preterm neonates were described and compared between the early and late caffeine treatment groups. Categorical variables were analyzed using the Pearson *χ* test, while continuous variables were analyzed using either Student's *t*-test or the Wilcoxon rank test.

The dataset was randomly divided into a training set and testing set at a ratio of 7:3. To make the most of the available information on complications and birth status in a relatively small sample, the researchers first established four comprehensive disease scores (Discore) by using least absolute shrinkage and selection operator (LASSO) regression to summarize the complex complications for the four outcomes of interest (BPD, IVH, ROP, and NEC) (excluding PVL due to non-convergence) (13). The risk factors included preeclampsia, eclampsia, fetal growth retardation, fetal distress, premature rupture of membrane, gestational diabetes mellitus, cholestasis, acute chorioamnionitis, placenta previa, placental abruption, antepartum hemorrhage, vaginitis, neonatal respiratory distress syndrome, neonatal infection, intrauterine infection, infectious pneumonia, septicemia, pulmonary arterial hypertension, patent ductus arteriosus, and intracerebral hemorrhage. To perform multivariable analysis, logistic regression was conducted after adjusting the Discore and clinically significant variables, including gestational age, birth weight, and history of birth asphyxia in the training set. Adjusted odds ratios and their 95% confidence intervals (CIs) were calculated. The receiver operating characteristic (ROC) curve and area under the curve (AUC) were plotted in the training and testing set to evaluate the predictive performance of logistic regression. All statistical analyses were conducted using R software (version 4.1.0), with a significance level of 0.05.

## 3. Results

### 3.1. Maternal and patient characteristics

A total of 452 preterm infants were included in this study, with 227 in the ECT group and 225 in the LCT group. The demographic characteristics of the mothers and preterm infants in the two groups were compared as shown in Table 1. The average maternal age was 32.0 ± 5.5 years, and there was no significant difference between the two groups (*P *= 0.439). There were no significant differences in the rates of natural conception or common perinatal complications between the two groups (*P *> 0.05), including preeclampsia, gestational diabetes, premature rupture of membrane, acute chorioamnionitis, placenta previa, placenta abruption, eclampsia, cholestasis, antepartum hemorrhage, and vaginitis. Additionally, there were no significant differences in the gender ratio of preterm neonates, the number of SGA neonates, or the incidence of fetal growth retardation, intracerebral hemorrhage, and distress between the ECT and LCT groups (*P *> 0.05). However, a higher proportion of preterm neonates with a gestational age of less than 28 weeks were found in the LCT group (*P *< 0.001), and the proportion of low-birth-weight neonates in the LCT group was also higher than that in the ECT group (*P *< 0.001). Notably, the incidence of PDA, intrauterine infection, septicemia, neonatal infection, infectious pneumonia, and ventilation rate was higher in the LCT group than that in the ECT group (*P *< 0.001). The preterm infants in the LCT group exhibited a longer duration of oxygen supplementation and invasive ventilation (*P *< 0.001). Further details of complications of maternal and preterm neonates are listed in Supplementary Table S1.

**Table 1:** The comparison of maternal and preterm neonatal characteristics between the two groups.

Variables	Caffeine treatment		*t*/*χ*^2^	*P*
	Early (*N* = 227)	Late (*N* = 225)		
Maternal characteristics				
Age, mean (SD)	31.8 (5.3)	32.2 (5.6)	−0.774	0.439
Natural conception, *n* (%)	159 (70.0)	151 (67.1)	0.451	0.502
Preeclampsia, *n* (%)	45 (19.8)	41 (18.2)	0.099	0.754
Gestational diabetes, *n* (%)	62 (27.3)	48 (21.3)	1.881	0.170
Premature rupture of membrane, *n* (%)	41 (18.1)	54 (24.0)	2.056	0.152
Acute chorioamnionitis, *n* (%)	19 (8.4)	25 (11.1)	0.679	0.410
Placenta previa, *n* (%)	18 (7.9)	12 (5.3)	0.846	0.358
Placental abruption, *n* (%)	13 (5.7)	13 (5.8)	0.000	1.000
Eclampsia, *n* (%)	3 (1.3)	1 (0.4)	–	0.623 (Fisher exact test)
Cholestasis, *n* (%)	6 (2.6)	10 (4.4)	0.611	0.434
Antepartum hemorrhage, *n* (%)	5 (2.2)	1 (0.4)	–	0.216 (Fisher exact test)
Vaginitis, *n* (%)	9 (4.0)	8 (3.6)	0.000	1.000
Neonates characteristics				
Gender ratio, M/F	135/92	127/98	0.425	0.514
Gestational age, *n* (%)			34.272	<0.001
<28 weeks	24 (10.6)	69 (30.7)		
≥28, <32 weeks	146 (64.3)	130 (57.8)		
≥32, <37 weeks	57 (25.1)	26 (11.5)		
Birth weight, *n* (%)			19.638	<0.001
<1,500 g	136 (59.9)	178 (79.1)		
≥1,500, <2,500 g	91 (40.1)	47 (20.9)		
Birth asphyxia, *n* (%)	45 (19.8)	75 (33.3)	10.576	0.001
SGA, *n* (%)	37 (16.3)	31 (13.8)	0.562	0.453
NRDS, *n* (%)	169 (74.4)	175 (77.8)	0.518	0.472
PDA, *n* (%)	24 (10.6)	51 (22.7)	11.084	<0.001
PH, *n* (%)	5 (2.2)	13 (5.8)	2.900	0.089
Intrauterine infection, *n* (%)	46 (20.3)	79 (35.1)	11.719	<0.001
Septicemia, *n* (%)	24 (10.6)	46 (20.4)	7.676	0.006
Fetal growth retardation, *n* (%)	20 (8.8)	21 (9.3)	0.001	0.976
Fetal distress, *n* (%)	26 (11.5)	24 (10.7)	0.014	0.907
Neonatal infection, *n* (%)	176 (77.5)	196 (8.7)	6.474	0.011
Infectious pneumonia, *n* (%)	163 (71.8)	201 (89.3)	21.037	<0.001
Intracerebral hemorrhage, *n* (%)	8 (3.5)	16 (7.1)	2.222	0.136
Duration of oxygen supplementation, median (Q1, Q3), days	25 (12, 38.5)	39 (26, 55)	35,110.5	<0.001
Ventilation	62 (27.3)	158 (70.2)	81.572	<0.001
Duration of invasive ventilation, median (Q1, Q3), days	3 (2, 5) (*n* = 62)	6 (4, 10) (*n* = 158)	7,283	<0.001

SGA, small for gestational age; NRDS, neonatal respiratory distress syndrome; PDA, patent ductus arteriosus; PH, pulmonary hypertension.

### 3.2. Association between early caffeine treatment and incidence of ORDIN in preterm neonates

The differences in GA, BW, and the incidence of PDA, intrauterine infection, and septicemia between the ECT and LCT groups could potentially confound the analysis results regarding the outcomes of caffeine treatment. The incidence of ORDIN was compared among different GA/BW subgroups of patients. The patients were classified into three subgroups based on GA: (1) extremely preterm birth subgroup (GA < 28 weeks, 93 cases); (2) very preterm birth subgroup (28 weeks ≤ GA < 32 weeks, 276 cases); and (3) moderate to late preterm (32 weeks ≤ GA <37 weeks, 83 cases). Table 2 presents the results of this analysis. Among the extremely preterm subgroup, patients who received early caffeine treatment had a lower incidence of PIVH and ROP (five cases, 20.1%; 17 cases, 70.8%, respectively) compared to that of those who received late caffeine treatment (33 cases, 47.8%; 62 cases, 89.9%, respectively). In the very preterm group, patients who received early caffeine treatment had a decreased incidence of BPD (by 0.69-fold of LCT) and PIVH (by 0.40-fold of LCT) compared to that of those with late caffeine treatment (*P *< 0.05, Table 2). The moderate to late preterm infants who received either early or late caffeine treatment exhibited a similar incidence of BPD, PIVH, PVL, ROP, and NEC between the two groups (*P *> 0.05, Table 2).

**Table 2:** Comparisons of caffeine treatment outcomes to ORDIN by the GA subgroup.

Variables, GA subgroup	Caffeine treatment		*χ* ^2^	*P*
	Early (227 cases)	Late (225 cases)		
BPD, case (%)				
Extremely preterm	22 (91.7)	68 (98.6)	Fisher's exact test	0.162
Very preterm	64 (43.8)	82 (63.1)	9.461	0.002
Moderate to late preterm	1 (1.8)	2 (7.7)	Fisher's exact test	0.531
PIVH, case (%)				
Extremely preterm	5 (20.1)	33 (47.8)	5.369	0.02
Very preterm	12 (9.0)	29 (22.3)	10.792	0.001
Moderate to late preterm	4 (7.0)	2 (8.3)	0.000	1.000
PVL, case (%)				
Extremely preterm	0 (0)	5 (7.2)	0.69	0.406
Very preterm	2 (1.4)	3 (2.3)	0.017	0.896
Moderate to late preterm	1 (1.8)	0 (0)	Fisher's exact test	1.000
ROP, case (%)				
Extremely preterm	17 (70.8)	62 (89.9)	5.038	0.025
Very preterm	94 (64.4)	85 (65.4)	0.030	0.862
Moderate to late preterm	15 (26.3)	4 (15.4)	1.209	0.272
NEC, case (%)				
Extremely preterm	4 (16.7)	15 (21.7)	0.056	0.813
Very preterm	23 (15.8)	18 (13.8)	0.198	0.657
Moderate to late preterm	5 (8.8)	3 (11.5)	0.000	1.000

Table 3 shows that among VLBW preterm infants with BW < 1,500 g, those who received early caffeine treatment had a low incidence of BPD (ECT: 55.9%, LCT: 80.9, *P *= 0.000), ROP (ECT: 69.9%, LCT: 79.8%, *P *= 0.043), and PIVH (ECT: 11.8%, LCT: 33.1%, *P *= 0.000). However, there were no significant differences in the incidence of PVL (ECT: 1.5%, LCT: 4.5%, *P *= 0.235) and NEC (ECT: 15.4%, LCT: 16.8%, *P *= 0.737) between the two groups. For infants with a birth weight between 1,500 g and 2,500 g, the incidence of the indicated terms of ORDIN was comparable between the ECT and LCT groups (*P *> 005, Table 3).

**Table 3:** Comparisons of caffeine treatment outcomes to ORDIN by the BW subgroup.

Variables, BW subgroup	Caffeine treatment		*t*/*χ*^2^	*P*
	Early (227 cases)	Late (225 cases)		
BPD, case (%)				
BW < 1,500 g	76 (55.9)	144 (80.9)	21.826	<0.001
1,500 g ≤ BW < 2,500 g	11 (12.1)	8 (17.0)	0.288	0.592
PIVH, case (%)				
BW < 1,500 g	16 (11.8)	59 (33.1)	19.387	0.000
1,500 g ≤ BW < 2,500 g	5 (5.5)	5 (10.6)	0.575	0.448
PVL, case (%)				
BW < 1,500 g	2 (1.5)	8 (4.5)	1.411	0.235
1,500 g ≤ BW < 2,500 g	1 (1.1)	0 (0)	Fisher's exact test	1.000
ROP, case (%)				
BW < 1,500 g	95 (69.9)	142 (79.8)	4.101	0.043
1,500 g ≤ BW < 2,500 g	31 (34.1)	9 (19.1)	3.350	0.067
NEC, cases (%)				
BW < 1,500 g	21 (15.4)	30 (16.8)	0.113	0.737
1,500 g ≤ BW < 2,500 g	11 (12.1)	6 (12.81)	0.013	0.909

To reduce the impact of confounding factors, we next performed a Discore analysis and logistic regression analysis. The Discore analysis identified the most important risk factors for the occurrence of BPD, PIVH, ROP, PVL, and NEC in preterm infants. The results of logistic regression analysis were adjusted for these identified confounding factors.

### 3.3. Discores for BPD, IVH, ROP, and NEC

Supplementary Figure S1 shows the process of parameter selection for lasso regression. However, due to non-convergence, PVL was excluded in the lasso and logistic regression. By using 10-fold validation, the tuning parameters were estimated as 0.023, 0.016, 0.014, and 0.024 for BPD, PIVH, ROP, and NEC, respectively. Under these parameters, important factors were automatically selected, and the Discores for BPD, PIVH, and ROP can be calculated as follows.

$$\begin{array}{rl} \mathrm{Discor}e_{\mathrm{BPD}}= & -2.682-1.097\times \mathrm{ECLAM}-0.262\times \mathrm{FGR}+0.015\\ & \times \mathrm{FIUD}-0.161\times \mathrm{ICP}+0.176\times \mathrm{AcuteHCA}\\ & -0.207\times \mathrm{HEMORRHAGE}+1.588\times \mathrm{NRDS}\\ & +0.399\times \mathrm{Infection}+0.323\times \mathrm{IAI}+1.388\\ & \times \mathrm{Pneumonia}+0.257\times \mathrm{Septicemia}+1.140\\ & \times \mathrm{PDA} \end{array}$$

$$\begin{array}{rl} \mathrm{Discor}e_{\mathrm{PIVH}}= & -4.043-0.078\times \mathrm{PE}-0.600\times \mathrm{FGR}-0.229\\ & \times \mathrm{FIUD}+0.067\times \mathrm{PROM}-0.724\times \mathrm{ICP}-0.850\\ & \times \mathrm{AcuteHCA}-2.158\times \mathrm{PLPREVIA}-0.578\\ & \times \mathrm{HEMORRHAGE}+1.918\times \mathrm{NRDS}+0.393\\ & \times \mathrm{Infection}+0.958\times \mathrm{Pneumonia}-0.064\\ & \times \mathrm{Septicemia}-0.405\times \mathrm{PH}+0.361\times \mathrm{PDA}\\ & +1.021\times \mathrm{ICH} \end{array}$$

$$\begin{array}{rl} \mathrm{Discor}e_{\mathrm{ROP}}= & -2.749+0.425\times \mathrm{PE}\text{-}1.517\times \mathrm{ECLAM}\\ & +0.145\times \mathrm{FGR}+0.359\times \mathrm{FIUD}+0.660\times \mathrm{PROM}\\ & -0.753\times \mathrm{AcuteHCA}-0.993\times \mathrm{PLPREVIA}\\ & -0.050\times \mathrm{PLABRUPT}-0.880\times \mathrm{VAGINOSIS}\\ & +3.640\times \mathrm{NRDS}-0.003\times \mathrm{Infection}+0.465\\ & \times \mathrm{Pneumonia}+0.040\times \mathrm{PH}+0.029\times \mathrm{PDA} \end{array}$$

$$\mathrm{Discor}e_{\mathrm{NEC}}=-2.749+0.3^{-15}\times \mathrm{PH}$$

The Discore for NEC showed almost no variation between the two groups, likely due to a very small coefficient for PH. However, the indices for the other three outcomes differed significantly between the two treatment groups (*P *< 0.05).

### 3.4. Logistic regression

Table 4 presents the result of the single-variable and multivariable analysis using logistic regression in the training set. After adjusting gestational age, birth weight, asphyxia, and Discore, we found that only the risk of PIVH in the ECT group was significantly lower than that in the LCT group (OR = 0.407, 95% CI, 0.188, 0.846).

**Table 4:** Effects of early administration of caffeine on ORDIN.

Outcomes	Caffeine group, *n* (%)		Odds ratio (95% CI)	
	Early (*n* = 143)	Late (*n* = 165)	Unadjusted	Adjusted^a^
BPD	54 (38)	113 (68)	0.279 (0.173, 0.445)	0.694 (0.350, 1.389)
PIVH	13 (9)	49 (30)	0.237 (0.118, 0.446)	0.407 (0.188, 0.846)
ROP	76 (53)	114 (69)	0.507 (0.317, 0.807)	0.697 (0.322, 1.501)
NEC	24 (17)	23 (14)	1.245 (0.667, 2.328)	1.428 (0.730, 2.811)

^a^ Adjusting with gestational age, birth weight, asphyxia, and Discore.

**Figure 1:**
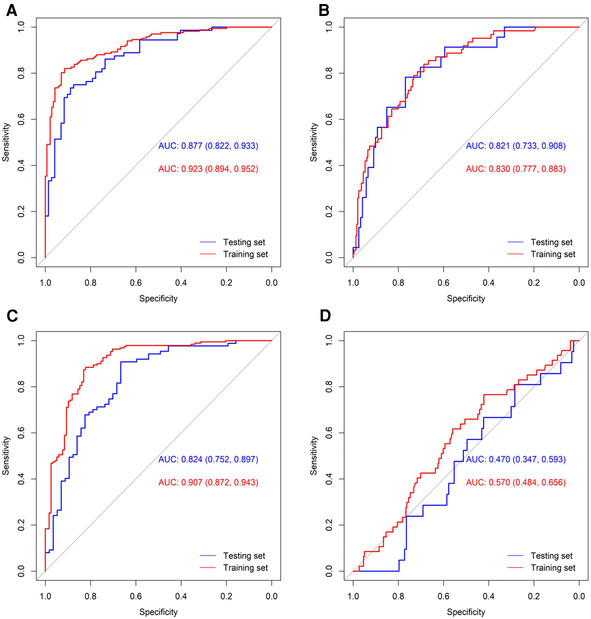
ROC curves of the multivariable models for the four outcomes. ROC curves of BPD (**A**), PIVH (**B**), ROP (**C**), and NEC (**D**) were shown.

Figure 1 displays the ROC curve of the four models used in the multivariable analysis. For BPD, the AUC under the training and testing set were 0.923 (0.894, 0.952) and 0.877 (0.822, 0.933), respectively. The AUC for PIVH under the training and testing set were 0.830 (0.777, 0.883) and 0.821 (0.733, 0.908), respectively. And the AUC for ROP under the training and testing set was 0.907 (0.872, 0.943) and 0.824 (0.752, 0.897), respectively. However, the factors included in the multivariable model could not properly predict the risk of NEC, with a very low AUC (both <0.600).

### 3.5. Sensitivity analysis of multiple factors

We conducted a sensitivity analysis (Supplementary Table S2) by including the duration of invasive ventilation and oxygen supplementation in the primary models and compared the coefficients and model fit. The model comparison (*P* values) for PIVH, ROP, and NEC showed that the factors of ventilation and oxygen supplementation did not significantly affect the fit of the primary model. However, the coefficient for BPD increased after adding these factors (*P *< 0.001), suggesting an impact on the fit of the primary model. Nevertheless, the 95% CI for the odds ratio indicated no significant association between early caffeine treatment and the incidence of BPD. Importantly, the 95% CI for the odds ratio revealed that early caffeine treatment only reduced the risk of PIVH, consistent with the findings in Table 4.

### 3.6. Treatment time exploration

**Figure 2:**
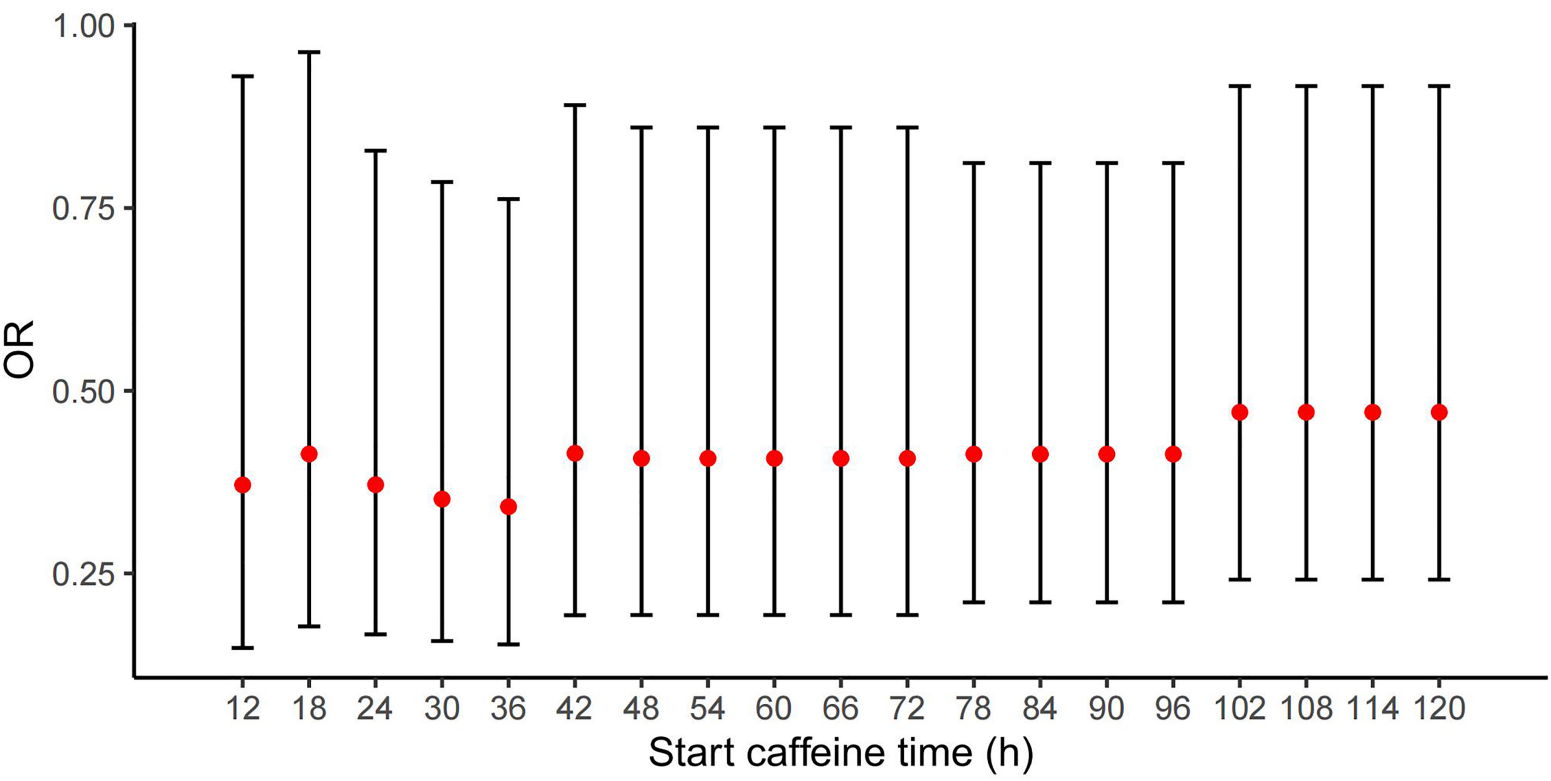
Effects of ECT under different initiating caffeine time definitions.

To further assess the impact of treatment time on the PIVH risk, we redefined the start time of ECT as 12 h, 18 h, and up to 120 h. We used the same multivariable models as in the previous section to examine the effects of ECT on PIVH incidence risk under different time definitions (Figure 2). Our results showed that early caffeine treatment could significantly reduce the risk of PIVH within the defined period. We set the cutoff time as 36 h after birth since the OR at this point was the smallest. However, we did not observe a significant difference between *t* other time points and the cutoff time point (*P *> 0.05). Despite smaller ORs being observed under the definition of 24, 30, and 36 h after delivery, *f* larger sample sizes or randomized controlled trials are necessary to determine the optimized initiating time point of caffeine treatment.

## 4. Discussion

In this retrospective study, the LASSO regression model demonstrated that early caffeine treatment was associated with a decrease in PIVH, but no other terms of ORDIN, after adjusting with gestational age, birth weight, asphyxia, and Discore. Additionally, ROC curves of multivariable analysis further illustrated that early caffeine treatment was linked to a reduced risk of BPD, PIVH, and ROP in preterm infants.

Our univariable logistic analysis indicated that early caffeine treatment was associated with a low risk of BPD. However, after adjusting with factors including gestational age, birth weight, asphyxia, and Discore in the multivariable regression analysis, this association was not supported. The results of subgroup analyses, which showed early caffeine treatment reduced the incidence of BPD in preterm infants, especially in VLBW or very preterm infants, suggest that the confounding factors in the retrospective study greatly influenced the evaluation of caffeine treatment with different initiation times. Previous research has also reported that early caffeine administration can reduce the risk of BPD in infants <29 weeks' GA, with an adjusted OR of 0.61 (95% CI, 0.45–0.81) (14). However, the adjusted factors for regression analysis and the GA of participants in that study differ from ours, which may explain the discrepancies between the two studies.

Preterm infants are particularly vulnerable to brain injuries due to the immaturity of their brains, with the “periventricular” area being critically susceptible. This area is at high risk of injury because of the dense network of thin and fragile capillaries that can easily be damaged. When these vessels rupture, it can lead to periventricular hemorrhage, which can develop into PIVH. PIVH mostly occurs in infants who are born before 33 weeks of GA (1).

Our study showed that the incidence of PIVH decreased in extremely and very preterm infants who received caffeine early (<48 h after birth) compared to that of those who received late caffeine treatment. We also found that VLBW infants had a lower incidence of PIVH when they received caffeine treatment earlier. Furthermore, both univariable and multivariable regression analysis after adjusting indicated clinical factors strongly suggested that early caffeine treatment is associated with a lower risk of PIVH in preterm infants. Additionally, ROC analysis in our study further showed that early caffeine treatment could predict the incidence of PIVH in preterm infants.

Our results differ from a previous clinical trial where there was no significant difference in the occurrence rate of PIVH in preterm infants with a GA of 25–35 weeks between early and late caffeine treatment groups (15). This discrepancy might be due to the lack of subgroup analysis of GA in that clinical trial. Another randomized controlled trial enrolled a total of 90 neonates with BW from 1,250 g to 2,000 g, who were clinically diagnosed with respiratory distress syndrome, and showed that caffeine treatment (initial dose at 20 mg/kg followed with a maintenance dose at 10 mg/kg daily) significantly reduced the incidence of PIVH compared to the control group (16). Similarly, a meta-analysis reported that initiating caffeine treatment early significantly reduced the risk of PIVH in VLBW infants (17). These studies strongly suggest that early caffeine treatment prevents the development of PIVH in preterm infants.

Regarding the optimal initiating time of caffeine treatment, our findings suggest that a window between 12 h and 120 h after birth could significantly reduce the risk of PIVH, with smaller odds ratios between 24 h and 36 h. However, further research with a larger sample size or randomized controlled trials is necessary to determine the most effective timing for initiating caffeine treatment.

PVL is a complication that can arise from PIVH. However, in our study, we did not observe a significant difference in the gross incidence of PVL or in subgroups between the early and late caffeine treatment groups. This finding is consistent with a retrospective study reported by Yang et al. (18).

Our study showed that the extremely preterm and VLBW infants who received early caffeine treatment had a lower incidence of ROP. The incidence of ROP is inversely correlated with GA and BW and is more common in extremely preterm and VLBW infants (19). Caffeine has been shown to have a protective effect on ROP in a large prospective clinical phase III trial and was considered as a prophylactic use for ROP (20). Our study of ROC analysis suggests that early caffeine treatment is associated with a lower incidence of ROP. However, the adjusted OR was not significantly different between the early and late caffeine treatment groups. This finding is in line with a study by Lodha et al. (14).

The effect of early caffeine treatment on the risk of NEC remains controversial. Taha et al. (21) reported that preterm infants receiving early caffeine treatment (initiating within 2 days after birth) had a higher risk of NEC (OR = 1.41, 95% CI, 1.04–1.91) while a recent study showed that the incidence of NEC was comparable between early and late treatment groups (22). Our finding is in line with the latter study, as we found no association between early caffeine treatment and the risk of NEC in preterm infants. Notably, our study and the recent study with similar findings both included Asian preterm infants, while Taha et al.'s study included mostly White and Black infants. This suggests that the outcomes of caffeine treatment may be influenced by the ethnic background of the infants.

The pathogenic mechanisms of various terms of ORDIN are distinct from one another. For instance, BPD is characterized by insufficient vascular development (23), while PIVH is dominantly associated with fragile immature vessels in the germinal matrix due to rapid angiogenesis (24). Immature cerebrovascular development is a critical risk factor accounting for the incidence of PVL (25). ROP development in preterm infants includes two phases: postponement of retinal vascularization and subsequent exaggeration of new and abnormal vessel growth resulting from increased release of vascular endothelial growth factor (VEGF) (19). The abnormal development of intestinal microvasculature is an important pathogenetic factor for NEC (26).

The effects of caffeine on angiogenesis vary in different tissues/organs and diseases. Studies have shown that caffeine treatment can attenuate BPD by improving angiogenesis in the lungs. For instance, postnatal use of caffeine (20 mg/kg/day) significantly improved pulmonary microvasculature by regulating angiogenic gene expression in male mice, such as VEGF and hypoxia-inducible factor (HIF)-1alpha (27). AR are widely expressed in the retina and brain. Blocking of AR by caffeine can reduce choroidal neovascularization by suppressing inflammatory responses and angiogenesis in choroidal and retinal endothelial cell migration (28). Caffeine does not affect normal retinal vessel growth but can significantly attenuate oxygen-induced retinopathy by blocking hypoxia-induced abnormal angiogenesis and vaso-obliteration, providing experimental evidence of prevention and treatment of ROP (29). Thus, the distinct effects of caffeine on angiogenesis in different types of tissues might partially explain why there are no significant effects of early caffeine treatment on certain terms of ORDIN, such as PVL and NEC.

A limitation of this study is its retrospective nature, which makes it vulnerable to confounding factors. Gestational age and birth weight are known to affect the outcomes of caffeine treatment in preterm infants. To minimize their impact, we performed stratified analyses in different gestational age and birth weight subgroups and used logistic regression with multivariate analysis. After adjusting with gestational age, birth weight, asphyxia, and Discore, preterm infants in the ECT group only had decreased odds of PIVH but no other terms of ORDIN, indicating that factors such as asphyxia, PDA, intrauterine infection, and septicemia may also affect the outcomes of caffeine treatment.

In conclusion, our retrospective study suggests that early initiation of caffeine treatment in Chinese preterm infants is associated with a reduced risk of PIVH. However, in our retrospective study, the findings are limited by potential confounding factors. Therefore, future prospective investigations are needed to verify and elucidate the precise effects of early caffeine treatment on complications in preterm infants. Overall, our study provides insights into the potential benefits of early caffeine treatment for preterm infants in Chinese neonatal intensive care units.

## Data Availability

The original contributions presented in the study are included in the article/**Supplementary Material**, further inquiries can be directed to the corresponding authors.
