# Supplementary material for: A two-center retrospective study: association of early caffeine administration and oxygen radical diseases in neonatology in Chinese preterm neonates
Source: Front Pediatr. 2023 Jun 14;11:1158286. doi: 10.3389/fped.2023.1158286 (PMC10303785; doi:10.3389/fped.2023.1158286)
Supplement: Supplementary file 1 [file Table1.docx]

Supplementary Material

A Two-center Retrospective Study: Association of Early Caffeine Administration and Oxygen Radical Diseases in Neonatology in Chinese Preterm Neonates

Huiqing Ye^1,2^ ^†^, Liyang Bai^1^ ^†^, Manting Yang^1^ ^†^, Xiaoyuan Yang^1,2^, Maofei Zheng^1^, Xiaobing Zhong^2^, Lifen Yang^2^, Zhuanggui Chen^2*^, Xinqi Zhong^1*^

^1^Department of Neonatology, the Third Affiliated Hospital of Guangzhou Medical University, Guangzhou, Guangdong, China

^2^Department of Pediatrics, the Third Affiliated Hospital of SunYat-sen University, Guangzhou, Guangdong, China

^†^These authors contributed equally to this work and share first authorship

*** Correspondence:**Xinqi Zhong, [zhongxq2016@gzhmu.edu.cn](mailto:zhongxq2016@gzhmu.edu.cn);

Zhuanggui Chen, [chenzhuanggui@126.com](mailto:chenzhuanggui@126.com).

# Supplementary Figures and Tables

For more information on Supplementary Material and for details on the different file types accepted, please see [here](https://www.frontiersin.org/guidelines/author-guidelines#supplementary-material).

## Supplementary Figures


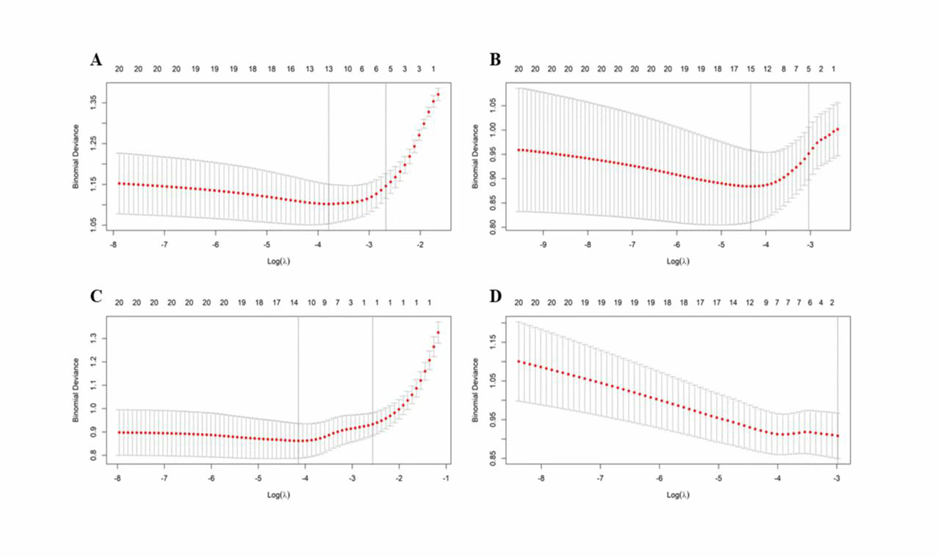


**Figure S1.** The parameter selection process of lasso regression. A, B, C, and D is the chart of BPD, PIVH, ROP, and NEC, respectively.

## 1.2 Supplementary Tables

| Table S1. Maternal and preterm neonates complications between two groups (n, %) | | | | |
| --- | --- | --- | --- | --- |
| Variables | Caffeine treatment | | c2 | *P* |
|  | Early | Late |  |  |
| Preeclampsia | 45 (19.8) | 41 (18.2) | 0.099 | 0.754 |
| Fetal growth retardation | 20 (8.8) | 21 (9.3) | 0.001 | 0.976 |
| Fetal distress | 26 (11.5) | 24 (10.7) | 0.014 | 0.907 |
| Premature rupture of membrane | 41 (18.1) | 54 (24.0) | 2.056 | 0.152 |
| Gestational diabetes mellitus | 62 (27.3) | 48 (21.3) | 1.881 | 0.170 |
| Acute chorioamnionitis | 19 (8.4) | 25 (11.1) | 0.679 | 0.410 |
| Placenta previa | 18 (7.9) | 12 (5.3) | 0.846 | 0.358 |
| Placental abruption | 13 (5.7) | 13 (5.8) | 0.000 | 1.000 |
| Neonatal respiratory distress syndrome | 169 (74.4) | 175 (77.8) | 0.518 | 0.472 |
| Intrauterine infection | 46 (20.3) | 79 (35.1) | 11.719 | < 0.001 |
| Septicemia | 24 (10.6) | 46 (20.4) | 7.676 | 0.006 |
| Pulmonary arterial hypertension | 5 (2.2) | 13 (5.8) | 2.900 | 0.089 |
| Patent ductus arteriosus | 24 (10.6) | 51 (22.7) | 11.084 | < 0.001 |
| *Fisher exact test. | | | | |
| Table S2. Sensitivity analysis for the multiple factors’ regression | | | | |
| Outcomes | Primary model  Odds ratio (95%CI) | Sensitivity model  Odds ratio (95%CI) | P value for model comparison | |
| BPD | 0.694 (0.350, 1.389) | 1.162 (0.466, 2.981) | <0.001 | |
| PIVH | 0.407 (0.188, 0.846) | 0.406 (0.185, 0.853) | 0.997 | |
| ROP | 0.697 (0.322, 1.501) | 0.642 (0.289, 1.412) | 0.245 | |
| NEC | 1.428 (0.730, 2.811) | 1.534 (0.767, 3.104) | 0.273 | |
